# Industrial Composting of Sewage Sludge: Study of the Bacteriome, Sanitation, and Antibiotic-Resistant Strains

**DOI:** 10.3389/fmicb.2021.784071

**Published:** 2021-12-24

**Authors:** Juan A. López-González, María J. Estrella-González, Rosario Lerma-Moliz, Macarena M. Jurado, Francisca Suárez-Estrella, María J. López

**Affiliations:** Unit of Microbiology, Department of Biology and Geology, CITE II-B, Agrifood Campus of International Excellence ceiA3, CIAIMBITAL, University of Almería, Almería, Spain

**Keywords:** biosolids, fecal contamination, metagenomics, antibiotic resistance, compost, enterobacteria, clostridia, fecal enterococci

## Abstract

Wastewater treatment generates a huge amount of sewage sludge, which is a source of environmental pollution. Among the alternatives for the management of this waste, industrial composting stands out as one of the most relevant. The objective of this study was to analyze the bacterial population linked to this process and to determine its effectiveness for the reduction, and even elimination, of microorganisms and pathogens present in these organic wastes. For this purpose, the bacteriome and the fecal bacteria contamination of samples from different sewage sludge industrial composting facilities were evaluated. In addition, fecal bacteria indicators and pathogens, such as *Salmonella*, were isolated from samples collected at key stages of the process and characterized for antibiotic resistance to macrolide, β-lactam, quinolone, and aminoglycoside families. 16S rRNA phylogeny data revealed that the process clearly evolved toward a prevalence of *Firmicutes* and *Actinobacteria* phyla, removing the fecal load. Moreover, antibiotic-resistant microorganisms present in the raw materials were reduced, since these were isolated only in the bio-oxidative phase. Therefore, industrial composting of sewage sludge results in a bio-safe final product suitable for use in a variety of applications.

## Introduction

The accelerated appearance of antibiotic resistance has become a global health problem (Abadi et al., 2019; Mobarki et al., 2019). In fact, it is estimated that by 2050 there will be 10 million deaths as a result of this problem (He et al., 2020). The situation is aggravated by the spread caused by the continuous generation of waste of anthropogenic origin (Karkman et al., 2018). These include, among others, sewage sludge, which contains heavy metals, organic compounds, pathogens (Lamastra et al., 2018), and traces of antibiotics that promote the emergence of antibiotic-resistant bacteria (ARGs) (Sun et al., 2019; Urra et al., 2019). After treatment, this waste can be used as fertilizer and for soil bioremediation (Eid et al., 2018), as well as to obtain energy and heat (Abuşoğlu et al., 2017). Therefore, a good management of these biosolids is crucial to avoiding the environmental and sanitary threats derived of the application of improperly treated sewage sludge (Kacprzak et al., 2017).

Current alternatives for the treatment of sewage sludge include composting, anaerobic digestion, and thermochemical processes, such as incineration, pyrolysis, or gasification (Raheem et al., 2018; Gao et al., 2020). Composting is considered one of the best options due to its sustainability and integration into circular bioeconomy concept, which is what the current European system is committed to Razza et al. (2018). This process generated a safe and stable bioproduct, the compost, which can be used as organic fertilizer (Soobhany et al., 2017). Moreover, the high temperatures reached during the process eliminate possible pathogens and, in addition, could reduce ARGs present in the raw materials (Zittel et al., 2020).

The culture techniques only estimate about 3% of the microbiota present in an environmental sample (Rubin, 2008). The development of “omics” as an alternative to traditional methods has allowed a deeper understanding of the microbiota present in a given habitat. Metagenomic analysis, through the use of bioinformatics tools, makes it possible to know even the non-culturable microbiota. In this way, it is possible to study in a more exhaustive way the microbial evolution that occurs during any environmental process, as it is the case of composting (Estrella-González et al., 2020b; Ma et al., 2020). Thanks to these tools, the presence of certain microbial groups can be effectively tested. This is particularly interesting since prokaryotes are mainly responsible for the degradation of toxic substances and the reduction of ARGs (Yadav and Kapley, 2019; Zhang et al., 2020). In this context, several authors relate through their research the efficacy of composting to remove ARGs (Su et al., 2015; Zhang et al., 2019; Tang et al., 2021; Zhu et al., 2021). Employing metagenomics for the analysis of swine manure composting, it has been reported that the host microbiota influences the depletion of these genes more than the environmental factors of the process itself (Zhang et al., 2018). Furthermore, in the disappearance of some genera such as *Acinetobacter* or *Pseudomonas* could also be related (Wu et al., 2020). Therefore, the possibility of corroborating these promising results on industrial composting of sewage sludge is of significant relevance. For this purpose, conventional cultivation techniques and culture-independent techniques can be successfully combined.

Based on the above, the main objectives of this work were (1) to analyze the evolution of the process in three industrial sewage sludge composting facilities using basic monitoring parameters; (2) to study the bacteriome of all processes; (3) to evaluate the fecal bacterial contamination levels during composting, through the study of indicator parameters, as well as human pathogens, using culture-dependent techniques; and (4) to characterize the antibiotic resistance through the industrial composting of a group of strains isolated from the positive results of the evaluation fecal load.

## Materials and Methods

### Sampling Treatment

Samples were collected from three industrial sewage sludge composting Spanish facilities located in the Southeast of Spain (SS1, SS2, and SS3). SS1 and SS2 operated with open air and turned windrows. On the other hand, SS3 used an in-vessel tunnel composting system with turning by augers. The composting process lasted for 3 months in all cases, with a bio-oxidative phase of 2 months for SS1 and SS2 and 1 month for SS3. The composting mixture consisted of sewage sludge + straw (1:1 v/v) for SS1, sewage sludge + pruning wastes (1:1 v/v) for SS2, and dried sewage sludge + pruning wastes (1:2 v/v) for SS3. All sewage sludge consisted of activated sludge. The different samples represented the most characteristic phases of the composting process. These were samples from raw material (RM), mesophilic phase (MES), thermophilic phase (TER) (>60°C), cooling phase (COOL), maturation phase (MAT), and final product (FP). At each stage of the process, 300-g subsamples were collected from different points and depths of the piles (with depths of 0.5 m, 1.5 m, and the bottom). These subsamples were then mixed to constitute 3 kg of representative sample. Samples for control parameter analysis were stored at −20°C in vacuum bags. Samples for analysis of biological parameters were fresh processed after sampling. For the study of carbon, nitrogen, and organic matter, the material was dried and ground up to a particle size <1 mm.

### Control Parameter Analysis

The parameters of control were carried out in all the samples. Moisture was determined by drying at 100°C for 24 h with 20 g of the sample. All the data were expressed on a dry-weight basis. The pH and electrical conductivity were analyzed from a 1:10 dilution of the samples in distilled water using Crison BASIC 30 (Crison Instruments, S.A., Barcelona, Spain). In the case of bulk density (BD), each sample was placed in a PVC cylinder of known dimensions. The weights before and after being dried in an oven at 60°C for 24 h were used to calculate the BD with the following expression: BD (g cm^−3^) = dry sample weight (g)/cylinder volume (cm^3^). The C/N ratio was obtained from the elemental determination of the carbon and nitrogen contents by combustion of the sample at 950°C using an Elementar Vario Micro CHNS (Elementar Analysensysteme GmbH, Hanau, Germany). The organic matter was determined by incineration at 550°C for 3.5 h.

### Fecal Contamination Evaluation

The bacterial fecal load was analyzed in SS1 using indicators of fecal contamination and human pathogens of interest in sewage sludge (total coliforms, fecal coliforms, *Escherichia coli*, fecal enterococci, sulfite-reducing clostridia, and presence of *Salmonella* and *Listeria*). For quantification, serial dilutions in 0.9% saline solution were prepared with the sample and inoculated in specific medium for each microbial group. The quantification of coliforms and fecal enterococci was performed using the Most Probable Number (MPN) method. In the case of coliforms, lactose broth (Scharlab, S.L., Barcelona, Spain) medium with bromocresol purple with the Durham tube was used. The temperature of incubation was different between total coliform, 37°C, and fecal coliform, 44.5°C. The presence of *E. coli* was determinate by plating on EMB agar (Panreac, ITW, Chicago, IL, United States) using the positive fecal coliform tubes. Rothe broth (Scharlab, S.L., Barcelona, Spain) was used to count fecal enterococci at 37°C. These were confirmed by Gram stain. The sulfite-reducing clostridia group was quantified using the method described by Huong et al. (2014), and after incubation at 37°C under anaerobic conditions, black colonies were counted. All these cultures were incubated for 24–48 h. The detection of *Salmonella* and *Listeria* was performed using the pre-enrichment technique by placing 25 g of sample in 225 ml of Buffered Peptone Water (AppliChem, ITW Reagents, IL, United States) for 24 h at 37°C. Then, in the case of *Salmonella*, 1 ml was added to Selenite Cystine broth tubes (Thermo Fisher Scientific, Waltham, MA, United States) and incubated for 18 h at 37°C. This culture was used to inoculate Hektoen agar (Panreac, ITW, IL, United States) plates by streaking, and after incubation for 24 h at 37°C, green colonies with a blackened central zone were confirmed on API 20E (biomerieux, Marcy-l’Étoile, France). For *Listeria*, Fraser broth (Merck, Darmstadt, Germany) was used to promote the growth of this pathogen. Suspected *Listeria* colonies were detected in PALCAM agar (Merck, Darmstadt, Germany), and then they were confirmed with an API test 07887Q (biomerieux, Marcy-l’Étoile, France). Two strains of *Listeria*, *L. innocua* ATCC 33090, and *L. monocytogenes* ATCC 13932, were used as controls in this biochemical characterization test.

### Detection of Listeria by qPCR

To correlate the results obtained for *Listeria* with culture technique, a qPCR was performed using the iQ-Check *Listeria* spp. Kit (Bio-Rad Laboratories, Inc., Hercules, CA, United States). Previously, pre-enrichment cultures were obtained by placing 25 g of the SS1 sample in *Listeria* Special Broth (LSB) medium (Bio-Rad Laboratories, Inc., Hercules, CA, United States) and incubated at 37°C for 24 h for pre-enrichment. Once the DNA was extracted from pre-enrichment culture according to the kit instructions, a real-time PCR system CFX Connect Real-Time (Bio-Rad Laboratories, Inc., Hercules, CA, United States) was used with the following thermal profile: 10 min at 95°C, 49 cycles of 15 s at 95°C, 20 s at 58°C, and 30 s at 72°C. The samples having a *Cq* value of 26 ≤ *Cq* ≤ 36 were given as positive for the FAM fluorophore. Apart from the kit positive control, the strains *L. innocua* ATCC 33090 and *L. monocytogenes* ATCC 13932 were used as additional controls.

### Antibiotic-Resistant Characterization

A collection of pure cultures was obtained from each positive result of the fecal evaluation and detection of the presence of pathogens described in section “Fecal Contamination Evaluation.” These strains were subjected to an evaluation of antibiotic resistance based on the method described by Kirby–Bauer (Bauer et al., 1966), which studies the sensitivity of a microorganism to an antibiotic or chemotherapeutical agent (antibiogram). For this purpose, disks impregnated with antibiotics (Thermo Fisher Scientific, Waltham, MA, United States) of the β-lactam, macrolide, quinolone, and aminoglycoside families at a known concentration were used against the growth of the strains of the collection obtained. First, a culture of each strain was obtained in nutrient broth (Panreac, ITW, IL, United States) and adjusted equivalent to 0.5 on the McFarland scale. Then, these cultures were mass inoculated with sterile swab in the surface of Mueller–Hinton (Panreac, ITW, IL, United States) and disks of the antibiotics were put in the plate. Three strains (*E. coli* ATCC 13706; *Salmonella enterica* ATCC 10708; *Enterococcus faecalis* ATCC 33186) lacking antibiotic resistance acquired were used as controls. After incubation for 18 h at 37°C, the growth inhibition halo was measured. The strains with no inhibition halo were considered resistant.

### Sequencing of the 16S rRNA Gene and Data Processing

DNA extraction was performed using the DNeasy PowerSoil DNA isolation kit (Qiagen N.V., Hilden, Germany). The sequencing was performed on a MiSeq PE300 run (Illumina Inc., San Diego, CA, United States) at AllGenetics and Biology SL (La Coruña, Spain). A total of 54 samples were processed corresponding to 3 composting plants × 6 samplings × 3 replicates. The primers used for the amplification of bacterial DNA for sequencing were as follows: Bakt 341F (5′CCT ACG GGN GGC WGC AG 3′) and Bakt 805R (5′ GAC TAC HVG GGT ATC TAA TCC 3′). These primers amplified the variable regions 3–4 (V3–V4) of the 16s rRNA gene with an expected size of 530 pb. Blank and negative controls were used during the process to check for possible cross-contamination and contamination problems during library construction, respectively. The quality of the demultiplexed FASTQ files was verified by FastQC software (Andrews, 2010). Paired-end assembly of the R1 and R2 reads was performed with FLASH (Magoč and Salzberg, 2011), with a minimum length overlap of 30 base pairs. Sequences were processed using the QIIME2 pipeline (Bolyen et al., 2019). In short, reads were imported, quality filtered, and dereplicated with the q2-dada2 option (Callahan et al., 2016). The processed sequences were used for all the downstream analyses. The database used was Silva 132 (for taxonomy assignment).

### Statistical Analysis

The parameters analyzed were performed in triplicate, using the mean for the presentation of the data. The physicochemical and fecal contamination parameters and biodiversity indices obtained were subjected to statistical analysis using Statgraphics Centurion XVIII.I (StatPoint Technologies Inc., Warrenton, VA, United States). Discriminant analysis was used to assess the adequacy of the classification of the composting facilities, in order to find simple equations for estimation of the development of stages of these composting processes from easily analyzable parameters. For this purpose, all physicochemical parameters and biodiversity indices measured were used. RStudio (RStudio Inc., AGPL v3) was used to represent correlation analyses, 16S rRNA phylogeny data, and boxplots for biodiversity indices.

### Data Availability

Sequences are stored in the MG-RAST public repository, available by Accession Number PRJNA769273. The whole datasets generated and analyzed during the current study are available from the corresponding author on reasonable request.

## Results

### Development of Industrial Composting of Sewage Sludge

**FIGURE 1:**
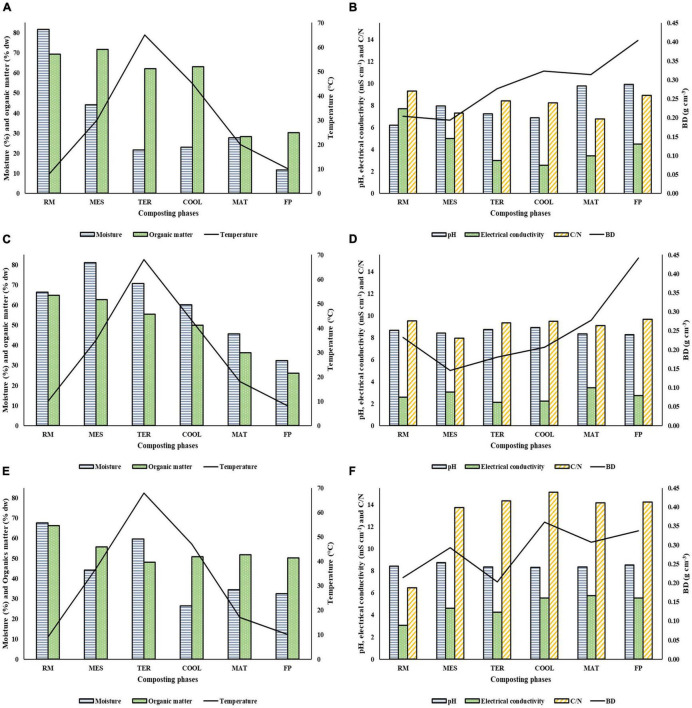
Control parameters analysis. Moisture (%), organic matter (% dw), and temperature (°C) are in Panels **(A,C,E)**; pH, electrical conductivity (mS cm^–1^), C/N, and BD (bulk density) (g cm^–3^) are in Panels **(B,D,F)**. Panels **(A,B)** represent SS1; Panels **(C,D)** represent SS2; Panels **(E,F)** represent SS3. SS1, SS2, and SS3 represent each of the sampled sewage sludge composting facilities.

The control parameters monitored during the composting processes are shown in Figures 1A–F. These parameters inform about the correct evolution of the process and included moisture, organic matter, temperature, pH, electrical conductivity, C/N ratio, and BD. The process started with values of moisture in RM belonging to SS2 and SS3 around 65–70% (66.37% in SS2 and 67.67% in SS3), while it was higher (81.65%) in SS1. This parameter decreased during composting in the three facilities analyzed. The organic matter reduced around 30–50% when the maturation phase was reached due to the mineralization. In all facilities, thermophilic conditions above 65°C were reached. In the case of pH, there were slight increases during the process up to values of 8.91, 8.26, and 8.52 units in SS1, SS2, and SS3, respectively, in the final compost. The electrical conductivity gradually decreased until the beginning of the maturation stage, from which it progressively increased up to final established values of 4.5, 2.72, and 5.52 mS cm^–1^ in SS1, SS2, and SS3, respectively. The highest data of the C/N ratio was obtained in SS3, in which at the end of the bio-oxidative phase, values above 13 were reached. The C/N ratio in SS1 and SS2 was lower than in SS3, and the value was less than 10 parts of carbon to 1 part of nitrogen remaining practically constant throughout the process. BD recorded some increases in the bio-oxidative phase and in the maturation phase, but it rose in the final product in all facilities reaching values of 0.40, 0.44, and 0.34 g cm^–3^ in SS1, SS2, and SS3, respectively.

### Fecal Contamination

The fecal contamination was evaluated in SS1, and the results are shown in Table 1. In the coliform group, there was an initial increase followed by a decrease and stabilization at the end of the process. The maximum levels of the three fecal indicators analyzed were reached at the MES phase, with log units of total coliforms and fecal coliforms of 5.81 and 2.45 log (cfu g^–1^), respectively. In this sampling phase, the entire fecal coliform count corresponded to *E. coli* species. The lowest microbial quantification was obtained in FP, with a value of 0.68 log units for both the total coliform and fecal coliform groups. *E. coli* was no longer detected since thermophilic conditions were reached. In the case of fecal enterococci, a significant increase was observed in the bio-oxidative phase (MES + TER) where counts above 3 log units were reached, decreasing afterward to reach 2.30 log units in the final composts. Sulfite-reducing clostridia showed a stabilization in the first stages of the process until a cooling phase was established. In that sampling, the higher counts [3.14 log (cfu g^–1^)] of this group were obtained. *Salmonella* was detected in the TER and COOL samplings. This pathogen was absent in the final product. *Listeria* was exempt in the whole process according to both culture techniques and qPCR results.

**TABLE 1:** Fecal contamination of SS1.

Composting phase	Total^1^ coliforms	Fecal^1^ coliforms	*E. coli* ^1^	Fecal enterococci^1^	Sulfite^2^-reducing clostridia	*Salmonella* ^3^	*Listeria* ^3^
RM	1.61	1.12	0	0.68	1.79	−	−
MES	5.81	2.45	2.45	3.27	1.88	−	−
TER	3.04	1.33	0	2.55	1.20	+	−
COOL	2.93	1.03	0	2.27	3.14	+	−
MAT	2.68	1.19	0	2.79	0.33	−	−
FP	0.68	0.68	0	2.30	0.90	−	−

*^1^Calculated with the most probable number (MPN) and expressed in log (cfu g^–1^).*

*^2^Calculated based on log (cfu g^–1^).*

*^3^Determination of the presence (+) or absence (-).*

### 16S rRNA Phylogeny Comparative

**FIGURE 2:**
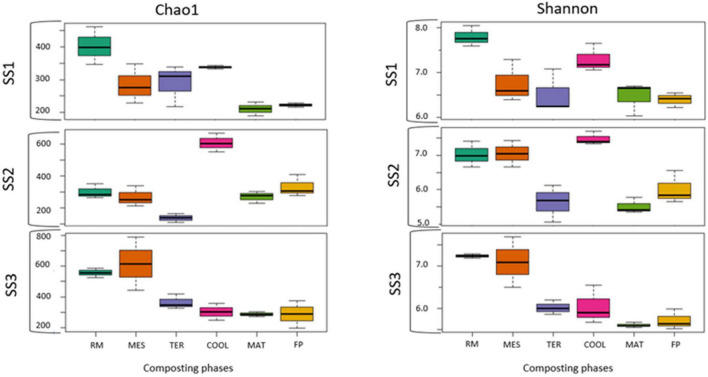
Boxplot of biodiversity indexes (Chao 1 and Shannon). SS1, SS2, and SS3 represent each of the sampled sewage sludge composting facilities.

The bacterial richness and abundance (Figure 2) of each sample were studied using the Chao1 and Shannon indexes, respectively. The maximum values for both indexes in SS1 and SS3 were found in the RM and COOL phases, of around 400–555 for richness and between 7 and 8 for the Shannon index. In SS2, the highest Chao1 value was obtained in the COOL stage (599) and the highest Shannon values were reached in RM (7.02) and COOL (7.5). The lowest values for richness and abundance in both SS1 and SS3 were observed in the final samples (MAT and FP). At these phases, the Chao1 index was around 180–195 and 280–285, in SS1 and SS3, respectively. With respect to the Shannon index, the data reached a maximum of 6.50 at maturation in SS1 and 5.72 in the final compost. The sample with the lowest richness and abundance for SS2 was TER in both indexes.

**FIGURE 3:**
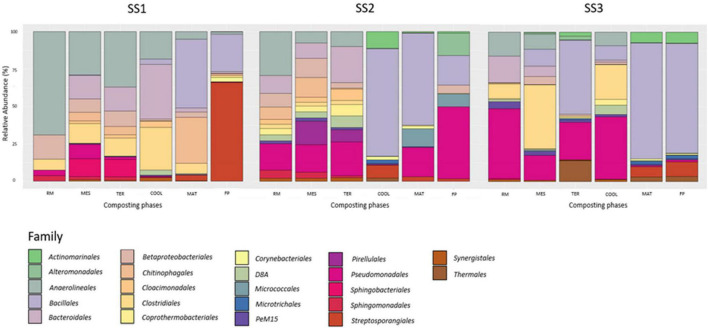
Relative abundance of bacterial families in compost samples from sludge sewage industrial-scale composting facilities. Raw material (RM), mesophilic phase (MES), thermophilic phase (TER), cooling phase (COOL), maturation (MAT), and final product (FP). The figure represents the percentage of presence of each bacterial family in the three sampled composting plants. SS1, SS2, and SS3 represent each of the sampled sewage sludge composting facilities.

Figure 3 shows the results of relative abundance of bacterial taxonomic assignation obtained after the 16S rRNA phylogeny analysis of each phase of the different industrial-scale sewage sludge composting processes. The three processes studied showed very different profiles. Globally, the bacteria at the dominant phylum level were *Firmicutes* (33.30%), *Proteobacteria* (20.70%), *Chloroflexi* (12.71%), *Bacteroidetes* (12.38%), and *Actinobacteria* (10.08%). However, the relative abundances of each sample were clearly different. In SS1, SS2, and SS3, the order Bacillales was present with percentages of 25.15, 19.67, and 73.50, respectively. In addition, in both SS1 and SS3, the order Streptosporangiales was detected. SS1 contained a percentage of 66.15 and 9.82% in SS3. In the case of SS2, the phylum *Proteobacteria* was the dominant phylum, divided into two families: Pseudomonadales (48.24%) and Alteromonadales (15.05%). Despite the ascribed differences between the facilities studied, there was a clear pattern of biodiversity loss in all cases. Once the thermophilic phase was overcome, the results showed a significant increase in the predominance of *Firmicutes*.

### Discriminant and Correlation Analysis

**FIGURE 4:**
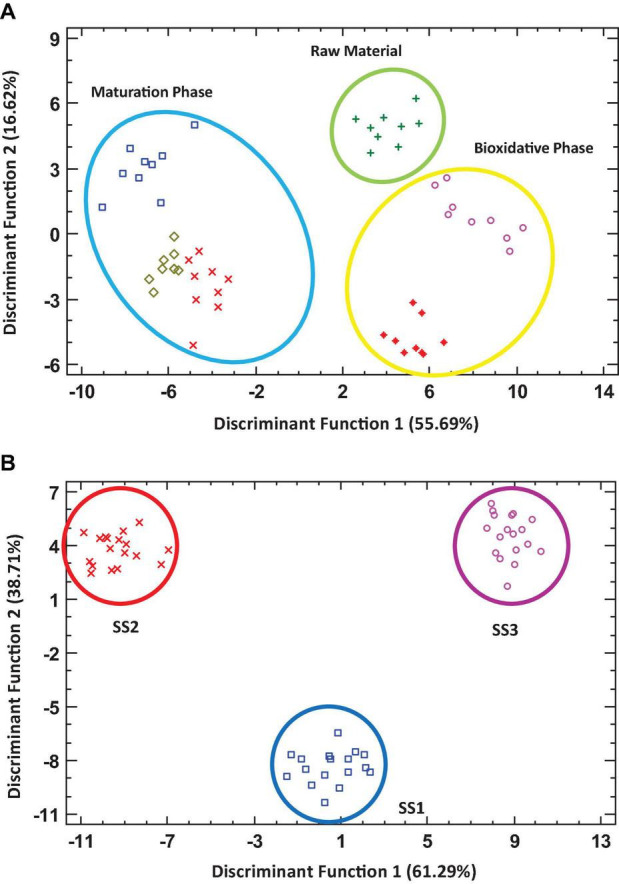
Discriminant analysis based on composting of different sewage sludge processes, taking into account the monitoring parameters, biodiversity indices, and abundance of majority families. Panel **(A)** represents the data as a function of composting phases; Panel **(B)** represents the data as a function of the facilities sampled.

Several statistical analyses were performed with the results obtained. Figure 4 shows two discriminant analyses. The first discriminant analysis (Figure 4A) presented two discriminant functions responsible for more than 72% of the variability of the data. The results were grouped into 3 data sets: one corresponding to the initial phase of the processes (RM), a second group formed by the data related to the bio-oxidative phase (MES + TER), and a third group including the data corresponding to the stabilization and humification phase (COOL + MAT + FP). Therefore, despite the heterogeneity of the data in relation to the starting materials and the working conditions of each facility, the data were appropriately grouped from an evolutionary point of view, establishing continuity and logical boundaries between the three main phases of the process. In the second discriminant analysis (Figure 4B), two discriminant functions responsible for 100% of the variability of the data were presented. According to these results, it is clear that the data related to monitoring parameters, biodiversity indices, and abundance of the majority families were grouped separately according to development of each facility.

**FIGURE 5:**
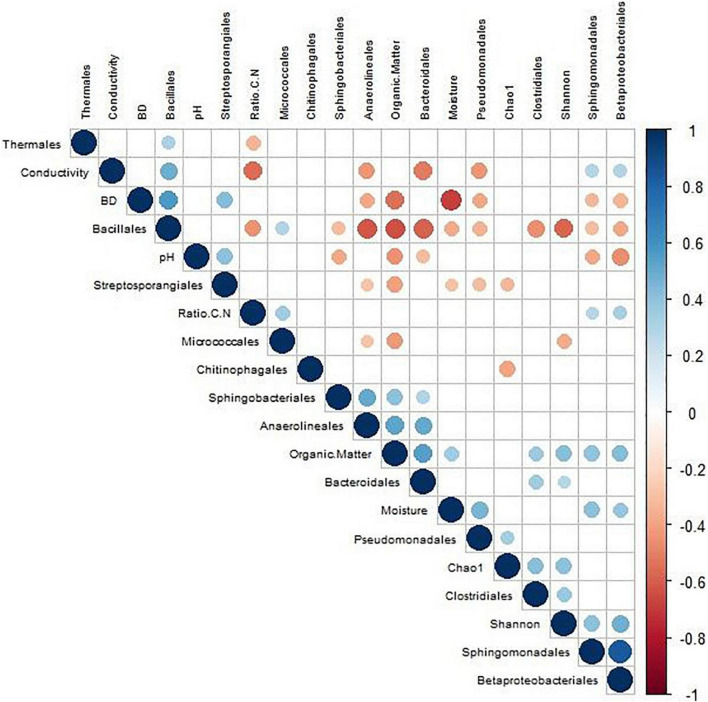
Pearson’s correlation analysis based on composting of different sewage sludge processes, taking into account the monitoring parameters, biodiversity indices, and most important families.

On the other hand, a Pearson correlation analysis was elaborated to check the relationship between the different parameters studied and the bacterial microbiota of the samples (Figure 5). The higher the correlation between the two parameters, the larger the size of the circle. The most significant values (*p* < 0.05) were the positive correlation between Sphingomonodales and Betaproteobacteriales (0.83) as well as Anaerolineales with respect to organic matter (0.55) and Bacteroidales (0.50). In contrast, the most significant negative correlations were those between Bacillales and Bacteroidales (−0.58), organic matter (−0.64), and Anaerolineales (−0.62) and the one presented by BD with respect to moisture (−0.69) and organic matter (−0.54).

### Antibiotic Susceptibility

The group of strains collected from fecal contamination analytics was composed of a total of 19 isolates, represented in Table 2. The vast majority were recorded before reaching thermophilic conditions in the compost piles (16 of the 19 isolates). The group with the highest representation in the collection was fecal coliforms, with up to 14 isolates, followed by 3 strains from the fecal enterococci group and two strains of *Salmonella*. There was a clear decrease in the number of strains once thermophilic conditions were established. In fact, only R19 was isolated in the maturation phase. Moreover, no strain was isolated from the final stage of the process.

**TABLE 2:**
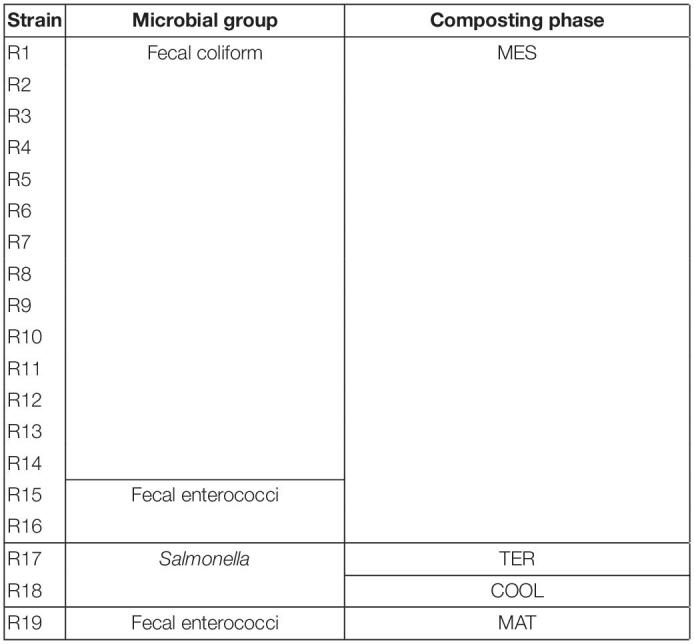
Group of strains isolated of each positive result of the fecal contamination during the composting process in SS1 with its codes.

**FIGURE 6:**
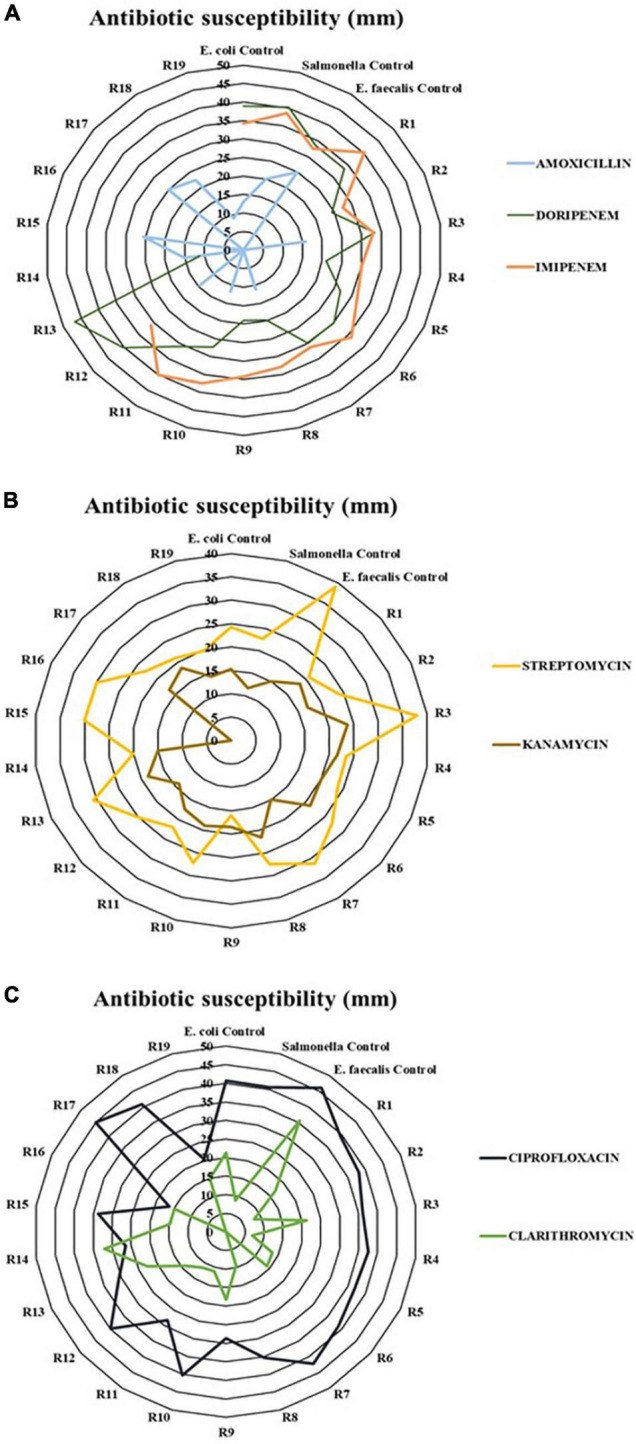
Antibiotic characterization of the strains obtained and the three controls used (*E. coli* ATCC 13706; *Salmonella* ATCC 10708; *E. faecalis* ATCC 33186). Panel (A) represents beta-lactam antibiotics; Panel (B) represents aminoglycoside antibiotics; and Panel (C) represents quinolone and macrolide antibiotics.

The results of the antibiotic susceptibility are represented in Figure 6. The antimicrobials ttable 2hat had a greater antibiotic effect and inhibited the growth of the majority of the strains were ciprofloxacin, imipenem, and streptomycin, with an approximate average of growth inhibition halo of 34, 34, and 25 mm, respectively. By contrast, amoxicillin was the less effective antibiotic given that 11 out of 19 strains were resistant to this compound. The isolates showed resistance to at least one antibiotic. However, the strain belonging to fecal enterococci, R16, showed resistance to azithromycin, kanamycin, and amoxicillin.

## Discussion

### Development of the Industrial Composting

Composting requires an exhaustive control of the different parameters that affect the process. This is necessary in order to obtain a final product with high quality that allows it to be used safely and efficiently (Siles-Castellano et al., 2020). In this sense, a moisture around 50–60% in the material is crucial to ensuring the correct proliferation of microorganisms (Jurado et al., 2020). The raw materials analyzed showed moisture values suitable for demands of the aerobic microbiota until they ended up at around 30% in SS2 and SS3 (Figure 1). However, in SS1 the final content of this parameter was too low, due to the lack of irrigation of the pile. This was a punctual fact since during the composting process, a correct humectation was provided. Regarding organic matter, a decrease at the start of the maturation phase, similar to that by Fialho et al. (2010), was observed. Thus, the percentages were according to the European Union regulation (EU, 2019). The temperature recorded during the process informs about the stabilization of the material and the degradation of the organic matter (Estrella-González et al., 2019). In all facilities, at the thermophilic phase the temperature was higher than 65°C. This is essential because temperatures above 50°C during, at least, 7 days ensure the sanitation of materials (Zhu et al., 2016). The pH in the compost obtained in all facilities was adequate for the agronomic use of the compost (Bernal et al., 2017; Azim et al., 2018). Based on Awasthi et al. (2014) and Chowdhury et al. (2015), an electrical conductivity of 4 mS cm^–1^ in the final product is appropriate. With the exception of SS2, in both SS1 and SS3, data around the above were recorded (Figures 1B,F). The C/N ratio affects directly to the process development, and the initial optimal C/N ratio is 25–30 (Estrella-González et al., 2020a). Nevertheless, the facilities analyzed started with a lower value of the C/N ratio. This is because sewage sludge has a high amount of nitrogen (Witter and Lopez-Real, 1987) and the initial mixtures were not ideal. These low C/N values can cause a loss of nitrogen during the process (Zhang et al., 2016). BD values close to 0.2–0.35 g cm^–3^ are considered correct for composting (Chang et al., 2019). In this sense, BD data of SS1, SS2, and SS3 complied with the above and showed the typical increase in that parameter while organic matter was being degraded.

### Fecal Indicators

The agronomic applications of the final products of the composting process make it necessary to ensure sanitation during the different phases. For this purpose, fecal indicators are adequate to determine the sanitary quality of the composts obtained (Manga et al., 2021). According to EU Regulation 2019/1009 (EU, 2019), *E. coli* or fecal enterococci counts must be less than 1,000 cfu g^–1^ [log (cfu g^–1^) < 3] in final products. Moreover, *Salmonella* has to be absent in 25 g of compost. The results of the evaluation of the fecal contamination, as shown in Table 2, demonstrated that the fecal indicators evaluated were well below the limit indicated by the legislation. In fact, *E. coli* was only detected in the mesophilic phase. The temperature reached once thermophilic conditions were established was not suitable for the proliferation of this species (Santos et al., 2020). In the case of fecal enterococci, which have higher survival capacity, the levels were above the coliform group. This group overcame the range of 1,000 cfu g^–1^ in the MES phase. Nevertheless, at the end of the process, the counts were lower than the threshold set by the regulation (EU, 2019). These results are consistent with the research of Ozdemir et al. (2020), in which the fecal enterococci load decreased due to increased temperature. With regard to sulfite-reducing clostridia, these are only able to proliferate in anaerobic habitats. Therefore, as composting is an aerobic process, it impeded the growth of this group. Matiz-Villamil et al. (2021) observed a similar trend to that recorded in the present study. These authors found that the counts of *Clostridium* spp. decreased due to the toxic effect that the oxygen had in this genus. This could explain the lower counts compared to the other indicators of fecal contamination. The detection of *Salmonella* in TER and COOL samplings was not as expected since these pathogens are unable to proliferate above 60°C (Fatunla et al., 2017). In addition to the effect of temperature, *Salmonella* can also be eliminated by the microbiota present in the pile (El Hayani et al., 2021). The addition of fresh material could have caused the detection of this pathogen at these stages. The presence of *Listeria* in sewage sludge is quite frequent (Lasaridi et al., 2018; Gholipour et al., 2020). In the case of SS1, *Listeria* was not detected by either cultivar or culture-independent techniques throughout the process. This genus includes two species pathogenic to humans, *L. ivanovii* and *L. monocytogenes* (Luque-Sastre et al., 2018). Thus, its absence in the final product would indicate good sanitization since it is not a microorganism with high thermotolerance (Bilung et al., 2018).

### 16S rRNA Phylogeny Comparative and Statistical Analysis

One of the main problems in comparing the metagenomics results obtained is the large number of bioinformatics tools available. This can lead to different results depending on the algorithms used to calculate the different biodiversity indices (Prodan et al., 2020). Despite this, coincidences can be observed in the data published by other authors. According to Li et al. (2019) in their study on the composting of pig manure with corn straw, both the richness and abundance of this process were higher than those of the composting processes studied. This indicates that the sewage sludge generated a more restrictive environment for microbiota proliferation, probably due to the limited access to the carbon present. In another composting process elaborated with poultry manure and rice straw (Zainudin et al., 2020), the values of the Shannon index oscillated in the same range to those presented by the three facilities studied. In accordance with the results of Lv et al. (2015) based on the stability of the mixture of sewage sludge and cattle manure, the abundance of the analyzed samples decreased as the process progressed, which supports the data obtained for the Shannon index. In the present study, the similarity between the values of MAT (5.87 Shannon index value) and FP (6.05 Shannon index value) of the statistical analysis indicated that most of the detected bacterial microbiota was maintained until the end of the process (Figure 2). This trend was also observed in the studies of López-González et al. (2015), in which the bacterial community structure was preserved in both the maturation phase and the compost.

According to Cai et al. (2018), it is usual to find mostly members of the phylum *Proteobacteria* in composts made from sewage sludge. These data coincide with those obtained in SS2 (Figure 3). The presence in the compost of populations belonging to the phylum *Actinobacteria*, as in the case of SS1, is indicative of stabilization and maturation of the materials generated. This is because *Actinobacteria* species participate in the biodegradation of recalcitrant compounds and lignocelluloses, thus playing an important role in the last phase of composting (Cao et al., 2021). Gram-negative *Bacteroidetes* are the group of anaerobic bacteria whose main function in the fermentation system is to break down macromolecules (such as proteins, starches, cellulose, and fibrous substances). The decrease in abundance of this group throughout the process is attributed to the reduction in the amounts of readily usable proteins and carbohydrates (Cai et al., 2018), but above all to the persistence of aerobic and thermophilic environments in the compost piles. The order Bacillales belongs to the phylum *Firmicutes* and plays an important role in lignocellulose degradation in these environments (Li et al., 2019), due to its high thermotolerance (Moreno et al., 2021). Thus, it was expected that as composting processes develop, this group would gain predominance. Indeed, this is what happened. This phylum was the majority in all facilities, especially in SS3. Moreover, most of the orders found in the different phases coincide with those described by Silva et al. (2016).

In order to achieve a correct interpretation of the data, it is not sufficient to read the results ascribed to the microbiome present throughout the industrial processes. Therefore, in the present study, a statistical analysis was integrated to evaluate the interaction between biological and physicochemical parameters (Figures 4, 5). These statistical data provided a true reflection of the evolution of composting. According to the information provided by discriminant analysis, it can be extracted that despite of the specificity of the evolution of the bacteriome in each facility, all of them present a development by which they end up converging in adequate stabilized products. This behavior has been previously described in industrial processes both from a physicochemical (Siles-Castellano et al., 2020) and microbiological (Estrella-González et al., 2020b) point of view. In this sense, Pearson’s correlations reflected an interesting relationship between the variables that mark the development of the composting process (organic matter and BD) with the disappearance of certain microbial groups that are not described as resident microbiota of the process but as part of untreated organic waste. Moreover, the opposite effect was produced in those groups that were found to be adapted to the process (Bacillales and Streptosporongiales). In summary, the behavior of the 16S rRNA phylogeny indicators used in the study show a loss of biodiversity associated with groups considered as part of the transient microbiota of the composting process (including microbiota associated with fecal contamination). This leads to an enrichment in resident populations of the compost, as it has already been reported by previous works of our research group (López-González et al., 2015). However, now the results possess the precision provided by the 16S rRNA phylogeny approach.

### Antibiotic Resistance

With the aim of corroborating that composting can control the spread of ARGs (Li et al., 2020; Wei et al., 2020a), the antibiotic resistance of a collection of bacterial isolates from compost was characterized in this study. Each strain, shown in Table 2, was isolated from the positive results of fecal evaluation. The majority of strains came from mesophilic phase sampling. However, when the thermophilic stage reached, there was a decrease in the number and diversity of isolates. This was clearly observed in the case of the fecal coliform group. These results are in concordance with the studies of Youngquist et al. (2016), Wang et al. (2018), and Arthurson (2020), where the microbial community, including pathogens, was reduced along the process because conditions were not suitable for the microbial growth. In sewage sludge, due to its anthropogenic origin, microbiota with ARGs and traces of antimicrobial compounds can be easily found (Wei et al., 2020b; Chen et al., 2021). Moreover, according to Wang et al. (2017), those bacteria that do not present resistance can acquire it through mobile genetic elements (MGEs). Therefore, without precise control of these wastes, the resistant bacteria present in them can be spread into the environment. Based on the results of the antibiogram, represented in Figure 6, strains isolated showed some resistance. In fact, amoxicillin was the least effective antibiotic since 11 of the 19 strains showed resistance to this compound. Liu et al. (2021) also reported the presence of microorganisms isolated of manures resistant to this antibiotic. The extensive use of amoxicillin in both humans and animals has increased resistance to this compound (Sodhi et al., 2021). On the other hand, most strains were sensitive to ciprofloxacin, streptomycin, and imipenem. However, none of the microorganisms were isolated from the final product. Thus, composting plays an important role in the elimination of ARGs. This is a possible approach to mitigate the damage of increasing antibacterial resistance.

## Conclusion

According to the results of the present study, the industrial composting of sewage sludge managed to generate a final product that complies with the hygienic–sanitary quality required by current legislation. During the bio-oxidative phase of the process, the values related to fecal contamination indicators carrying ARGs were significantly reduced, to the benefit of a resident microbiota adapted to the prevailing conditions. In the sewage sludge, there were numerous microorganisms of fecal origin that showed resistance to antibiotics routinely used in humans. Such resistances were detected mainly in microbial strains isolated from raw materials and the bio-oxidative phase of the process. Therefore, industrial-scale sewage sludge composting was shown to be an effective tool for the elimination of indicator strains of fecal contamination resistant to certain antibiotics in routine use. Thus, it contributed to the reduction of the spread of ARGs in environmental microbiomes, as well as the transfer of the same in the food chain.

## Data Availability Statement

The datasets presented in this study can be found in online repositories. The names of the repository/repositories and accession number(s) can be found below: https://www.ncbi.nlm.nih.gov/, PRJNA769273.

## Author Contributions

JL-G: formal analyses, investigation, supervision, and writing—original draft preparation. ME-G: investigation and writing—original draft preparation. RL-M: methodology, investigation, formal analyses, and writing—original draft preparation. MJ and FS-E: methodology and investigation. ML: methodology, visualization, supervision, and writing—review and editing. All authors contributed to the article and approved the submitted version.

## Conflict of Interest

The authors declare that the research was conducted in the absence of any commercial or financial relationships that could be construed as a potential conflict of interest.

## Publisher’s Note

All claims expressed in this article are solely those of the authors and do not necessarily represent those of their affiliated organizations, or those of the publisher, the editors and the reviewers. Any product that may be evaluated in this article, or claim that may be made by its manufacturer, is not guaranteed or endorsed by the publisher.
